# Immunity against *Ixodes scapularis* Salivary Proteins Expressed within 24 Hours of Attachment Thwarts Tick Feeding and Impairs *Borrelia* Transmission

**DOI:** 10.1371/journal.pone.0000451

**Published:** 2007-05-16

**Authors:** Sukanya Narasimhan, Kathleen DePonte, Nancy Marcantonio, Xianping Liang, Thomas E. Royce, Kenneth F. Nelson, Carmen J. Booth, Benjamin Koski, John F. Anderson, Fred Kantor, Erol Fikrig

**Affiliations:** 1 Section of Rheumatology, Department of Internal Medicine, Yale University School of Medicine, New Haven, Connecticut, United States of America; 2 Section of Allergy and Clinical Immunology, Department of Internal Medicine, Yale University School of Medicine, New Haven, Connecticut, United States of America; 3 Program in Computational Biology and Bioinformatics, Yale University, New Haven, Connecticut, United States of America; 4 Department of Biochemistry and Biophysics, Yale University, New Haven, Connecticut, United States of America; 5 Section of Comparative Medicine, Yale University School of Medicine, New Haven, Connecticut, United States of America; 6 Department of Entomology, Connecticut Agricultural Experiment Station, New Haven, Connecticut, United States of America; Massachusetts General Hospital and Harvard Medical School, United States of America

## Abstract

In North America, the black-legged tick, *Ixodes scapularis*, an obligate haematophagus arthropod, is a vector of several human pathogens including *Borrelia burgdorferi*, the Lyme disease agent. In this report, we show that the tick salivary gland transcriptome and proteome is dynamic and changes during the process of engorgement. We demonstrate, using a guinea pig model of *I. scapularis* feeding and *B. burgdorferi* transmission, that immunity directed against salivary proteins expressed in the first 24 h of tick attachment — and not later — is sufficient to evoke all the hallmarks of acquired tick-immunity, to thwart tick feeding and also to impair *Borrelia* transmission. Defining this subset of proteins will promote a mechanistic understanding of novel *I. scapularis* proteins critical for the initiation of tick feeding and for *Borrelia* transmission.

## Introduction

The black-legged tick *Ixodes scapularis* is a vector of *Borrelia burgdorferi*, the agent of Lyme disease [1] and of pathogens responsible for anaplasmosis [2], babesiosis [3], and tick-borne encephalitis [4]. *I. scapularis* nymphs transmit these pathogens during their 3–8 days of attachment [5] and feeding [6], [7]. The tick's salivary components facilitate microbial transmission [8], [9]. Defining tick proteins critical for feeding and transmission is essential for a molecular basis of new vaccines against tick-borne pathogens. Towards this goal, research efforts focused on salivary gland proteins expressed in ticks that fed for 3–4 days or longer, primarily due to the ease of obtaining protein and RNA from fed, compared with unfed, ticks [10], [11], [12]. Several tick salivary proteins with pharmacological activities that block host haemostatic cascades and immune responses have been identified in this way [11], [12], [13], [14], [15], [16], [17], [18], [19], [20], [21]. The potential of these proteins to serve as efficient vaccines to block feeding, however, has not yet been demonstrated. In recent studies, Subolesin, a tick protein identified from an *I. scapularis* embryonic cell-line [22], [23], and 64TRP [24] a recombinant version of a secreted salivary gland cement protein from *Rhipicephalus appendiculatus* have demonstrated potential as vaccines to impair tick infestation. Immunity against 64TRP, a potential broad-spectrum vaccine candidate was shown to also decrease the transmission efficiency of TBE by *I. ricinus* nymphs [25].

The research impetus to identify tick salivary proteins critical for feeding has been driven largely by the phenomenon of acquired tick-immunity, first described by William Trager [26]. Trager showed that guinea pigs repeatedly infested by *Dermacentor variabilis* larvae acquired immunity against ticks, which was characterized by cutaneous reactions marked by edema and inflammatory infiltrates that led to impaired engorgement, decreased tick weights and increased mortality. Studies since then have described the phenomenon of acquired tick-immunity in a wide variety of tick-host species and ascribed the phenomenon to the elaboration of host humoral and cellular responses to tick salivary antigens secreted into the feeding site [27], [28], [29]. Importantly, acquired tick-immunity also impaired the transmission of pathogens to the vertebrate host [27], [30]. Research efforts for several decades endeavored to identify the components of tick saliva that reacted with tick-immune sera with the search shifting in vain between unfed and fed ticks [12], [31], [32], [33], [34]. We now examine why proteins from unfed and fed ticks, despite their reactivity with tick-immune sera, demonstrated only a limited role in provoking tick-immunity. We offer evidence that the transcriptome and proteome of the tick salivary glands is dynamic during feeding and address the impact of host immunity against the 24 h tick salivary proteins on tick engorgement and *Borrelia* transmission.

## Results

### The I. scapularis salivary gland proteome at 24 h is different from that at 66 h of feeding

**Figure 1:**
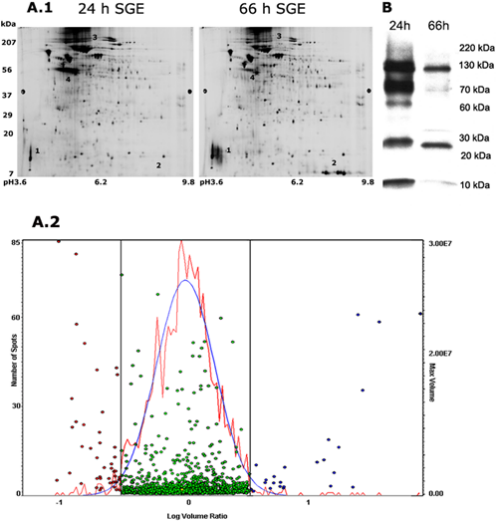
Proteome profile of 24 h and 66 h fed salivary glands. A.1. Differential 2D Fluorescence Gel Electrophoresis (DIGE) of 50 µg of proteins from 24 and 66 h fed salivary glands. Highlighted are proteins at positions 1 and 2 preferentially expressed at 66 h, while proteins at positions 3 and 4 are differentially expressed in 24 h salivary glands. A.2. Histogram of the global protein profiles plotted as a log ratio of spot intensity of Cy5/Cy3 (66 h/24 h) shows a set of proteins that are greater than 3 fold differentially expressed in 24 h (indicated by red circles) and 66 h (indicated by blue circles). Proteins less than 3 fold differentially expressed (indicated by green circles) are represented within the bell curve. B. A western blot of 5 µg of proteins isolated from 24 and 66 h fed salivary gland extracts probed with nymph-immune rabbit serum shows greater reactivity with proteins from the 24 h extracts than from the 66 h fed extracts.

We addressed two time points of feeding, 24 h representing an initial phase when feeding commences and 66 h representing a later phase of nymphal feeding prior to repletion. A comparative analysis of the protein profile of salivary glands from 24 h and 66 h fed *I. scapularis* nymphs was carried out by two-dimensional gel electrophoresis. While a majority of the proteins were represented in comparable amounts in both 24 and 66 h fed salivary glands, at least 6–10 proteins were differentially expressed (Fig 1A.1). We highlight a few of these including proteins corresponding to positions 3 and 4 which were uniquely represented in the 24 h salivary glands, and proteins at positions 1and 2 observed only in the 66 h salivary glands. Histogram of the global analysis as generated by the DeCyder (GE Healthcare, NJ) software package demonstrated that several proteins were greater than three fold differentially expressed between the two populations (Fig 1A.2). Further, western analysis of 24 and 66 h salivary gland proteins separated on a one dimension SDS-PAGE using *I. scapularis* nymph-immune rabbit serum showed a differential reaction with 24 and 66 h proteins (Fig 1B).

**Figure 2:**
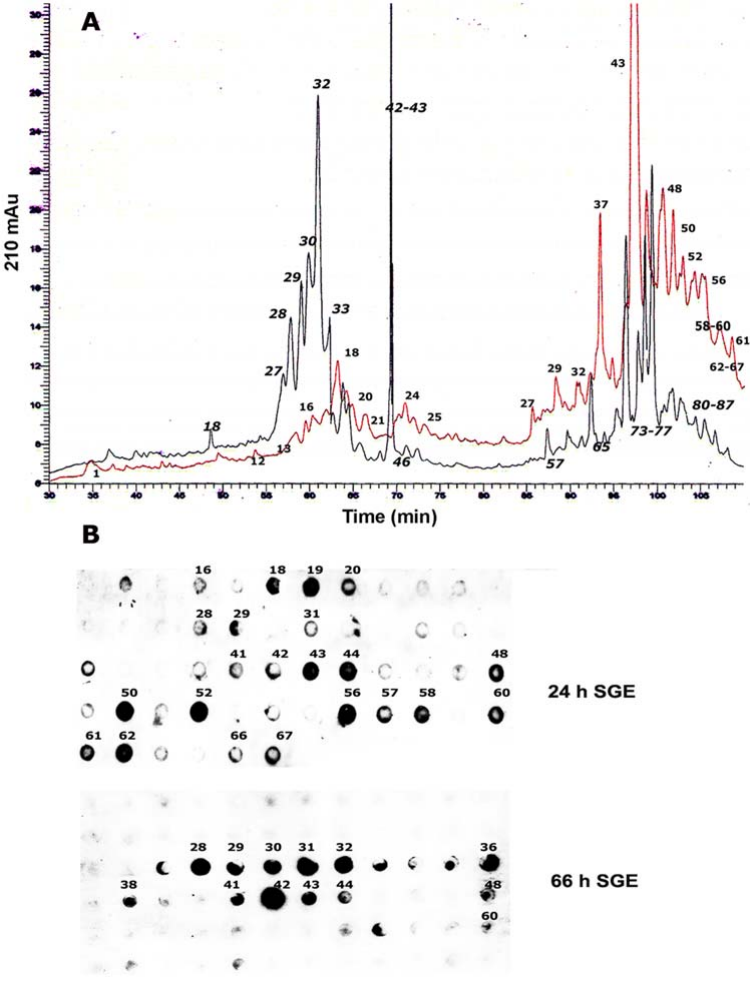
HPLC fractionation of 24h and 66 h fed salivary gland proteins. A. Chromatogram of the profiles of protein extracts from 24 h (shown in red) 66 h (shown in black) fed salivary glands fractionated on a C4 hydrophobic column shows differences in the protein composition of the two populations. Fraction numbers are indicated next to prominent protein peaks. B. A dot-blot analysis of the HPLC fractions using tick-immune rabbit serum demonstrates differential reactivity with 24 and 66 h protein fractions. Numbers next to the cross-reacting spots correspond to HPLC protein peaks.

Fractionation of total protein from 24 and 66 h salivary glands on a C4 reverse phase HPLC column also revealed differences in the profiles of the 24 and 66 h salivary glands (Fig 2A) and dot-blot western analysis of the fractions also showed that nymph-immune rabbit serum reacted differentially with the 24 and 66 h salivary proteins (Fig 2B).

### I. scapularis salivary gland transcriptome at 24 h is different from that at 66 h of feeding

**Figure 3:**
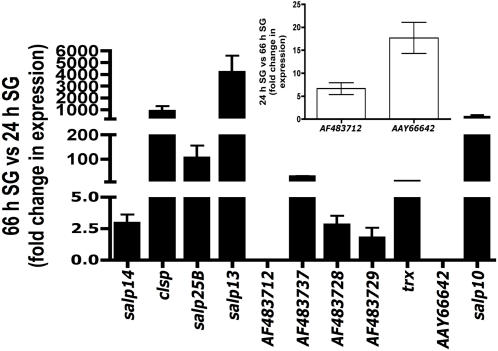
Quantitative RT-PCR demonstrates differential expression of salivary gland genes at 24 and 66 h of feeding. Relative quantitation of gene expression for selected set of salivary genes confirmed the observations made using the subset oligonucleotide array (Table 1). Fold change in gene expression at 66 h relative to 24 h. Error bars represent Mean fold change±SD. Expressions of most of the genes were increased at 66 h when compared to the levels at 24 h of feeding. The expression of at least 2 genes was increased at 24 h compared to that at 66 h (Inset) presented here as fold change in gene expression at 24 h relative to 66 h.

An oligonucleotide mini-array representing a subset of genes encoding for secreted salivary proteins was utilized to interrogate the expression profile of 24 and 66 h fed *I. scapularis* nymphs. Array elements that showed significant and consistent hybridization patterns in all replicate experiments are listed in Table 1. Differences in the expression profiles of several genes (Table 1) in the 24 and 66 h salivary glands showed that the tick transcriptome changes during the process of feeding. These observations were validated by quantitative RT-PCR of at least 10 selected salivary genes (Fig 3).

**Table 1:** Expression profiles of *Ixodes scapularis* salivary genes at 24 and 66 h of feeding.

Gene accession number	Putative function/homology	Ratio of 24 h/66 h pixel intensities	*P* value
AF483716	Protease inhibitor	−10	6e-5
AF483714	Putative protease inhibitor	+4	1e-2
AF483727	Putative serpin	+10	1e-2
AF483726	Protease inhibitor	+10	1e-2
AF483689	Secreted protease inhibitor	−3	1e-2
AF483712	Salp10-like	+10	6e-5
AF278575	Salp10	−20	1e-2
AF483719	Salp13 -like	1	6e-5
AF483720	-Salp13-like	−2	9e-3
AF209912	Salp13	−100	1e-2
AF483728	Secreted carboxypeptidase	−10	6e-5
AF483731	Secreted metalloprotease	1	6e-5
AF483740	Secreted 10.2 kDa	−3	6e-5
AF483688	Secreted convertase protease	−5	1e-3
AF483729	Transmembrane serine protease	−10	1e-3
AAY333959	Thoredoxin peroxidase	−20	1e-3
AF483690	Secreted protease	+7	5e-4
AF483743	Secreted lipocalin	−6	6e-5
AF483722	Lipocalin-like	1	6e-5
AF209920	Histamine binding protein	−20	1e-2
AF483718	Histamine-binding protein	+10	1e-2
AF209918	Salp25B-Histamine binding	−10	1e-2
AF483721	Salp25A-like	1	1e-1
AF209922	Salp25A-Histamine binding	−8	5e-4
AF483734	5.3 Kda protein/defense	1	6e-5
AF483737	Secreted protein	−10	6e-5
CD052513	Similar to Virilizer	+3	6e-5
AF209916	Salp17	−20	6e-5
AF483659	Salp14-like	1	1e-4
AF483662	Salp14-like	1	1e-4
AF483665	Salp14-like	+2	5e-4
AF483660	Salp14-like	+2	1e-2
AF483663	Salp14-like	1	1e-2
AAY66642	Secreted protein	+10	1e-2
AF209921	Salp14- anticoagulant	−4	1e-2
AF483663	Salp14-like	1	1e-2
AF483703	Secreted protein	−10	2e-4
AF483681	Secreted protein	−2	5e-4
AF483686	Secreted protein	−100	1e-2

Results presented as a ratio of the median of normalized intensities from Cy5 (24 h salivary gland) to the median of normalized intensities from the Cy3 (66 h salivary gland). P values calculated from the paired signed-rank test are shown.

### Salivary proteins expressed in the first 24 h of feeding are sufficient to elicit tick-immunity

**Figure 4:**
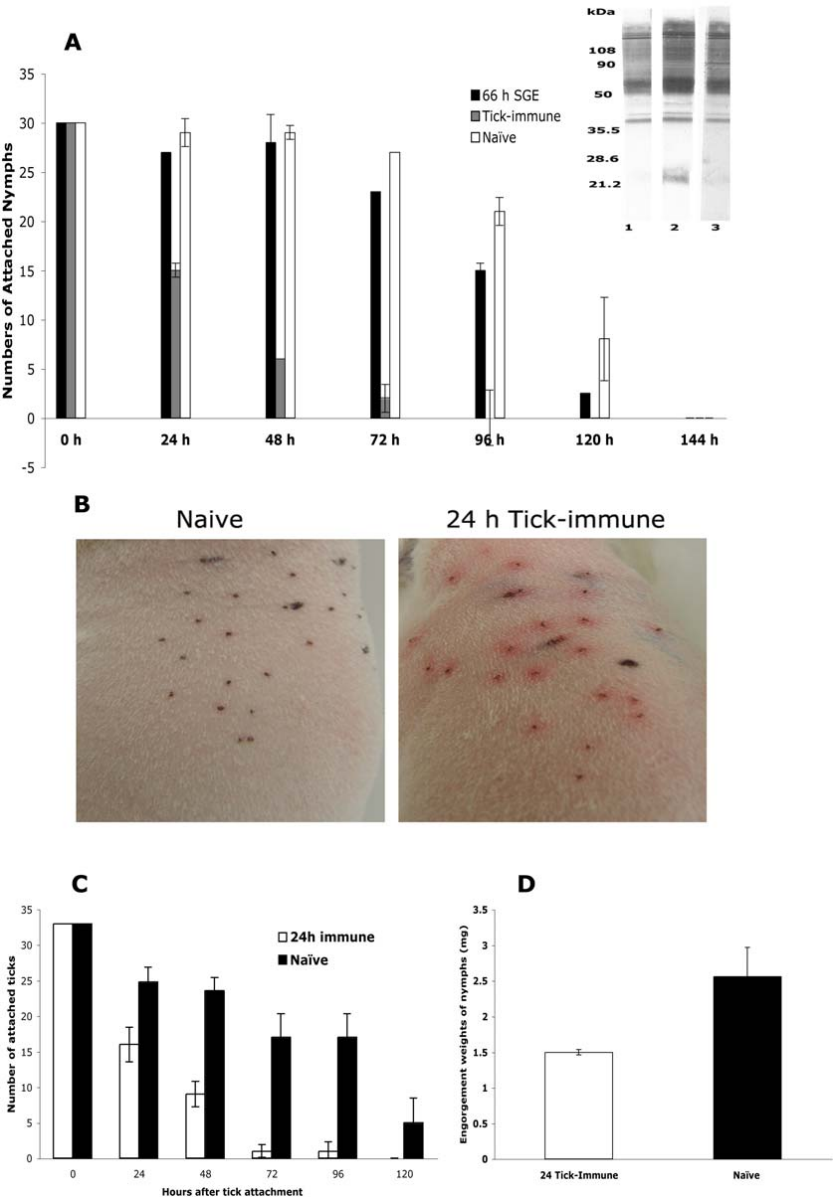
Proteins expressed within 24 h of tick feeding provoke tick-immunity. A. Guinea pigs challenged with *I. scapularis* nymphs subsequent to immunization with protein extracts from 66 h fed nymphal salivary gland extracts (66 h SGE); or after 3 tick infestations (Tick-immune). Naïve guinea pigs served as control. Inset. Immunoblots of tick salivary gland extracts (1 µg/lane) probed with immune sera from three 66 h SGE-immunized animals (lanes 1–3). Results are one representative of three replicate experiments. B. 24 h tick-immune guinea pigs when challenged with ticks showed erythema at the tick attachment sites within 24 h of tick attachment; no redness was apparent on naïve animals. C. Ticks feeding on 24 h tick-immune guinea pigs were rapidly rejected and the number of attached ticks decreased significantly (*P = 0.01*) within 48 h when compared to that on naïve animals. D. Ticks feeding on 24 h tick-immune animals showed decreased engorgement weights compared to ticks that engorged on naïve animals. Error bars represent Mean±SE.

Repeated tick-infestation of the vertebrate host provokes a vigorous immune response directed against tick salivary proteins and as a result, ticks feeding on tick-immune hosts are rejected within 24–48 h [27], [35], [36]. To begin to define *I. scapularis* salivary proteins that elicit tick-immunity, we immunized guinea pigs with salivary gland extracts from 66–72 h fed nymphs and confirmed by western analysis that the immunized guinea pig sera reacted with protein extracts from fed tick salivary glands (Fig 4A-inset). Upon challenge of the guinea pigs with nymphs, tick fall-off rates and engorgement weights on immunized animals were not significantly decreased when compared to that on naïve guinea pigs (Fig 4A)(*P = 0.12*). This raised the possibility that proteins contained in the 66–72 h salivary gland extracts may not play a critical role in provoking tick-immunity. Evidence that the tick salivary gland transcriptome and proteome is dynamic, prompted us to examine whether tick-immunity is perhaps directed against salivary proteins expressed in the first 24 h of attachment. To circumvent the tediousness involved in generating 24 h salivary gland extracts for immunization, we infested guinea pigs with *I. scapularis* nymphs and manually removed the nymphs 24 h after attachment to ensure that the guinea pig immune response was directed only against salivary proteins expressed in 24 h fed nymphs. This 24 h infestation was repeated at least 4 times, and these guinea pigs will henceforth be referred to as 24 h tick-immune animals. In contrast, guinea pigs that had been repeatedly infested with ticks that were allowed to feed normally [37] will be referred to as replete tick-immune animals. Each of the 24 h tick-immune guinea pigs, when challenged with 30 *I. scapularis* nymphs, demonstrated all the major hallmarks of acquired tick-immunity including erythema at the sites of tick attachment (Fig 4B), rapid rejection of ticks within 24–48 h (Fig 4C) and significantly lower tick engorgement weights (Fig 4D)(*P<0.05*) compared to ticks that fed on naïve guinea pigs. These observations suggested that host immunity directed against 24 h tick salivary proteins is sufficient to impair tick feeding and confirmed the posit that acquired tick-immunity is predominantly directed against 24 h salivary proteins and not against proteins expressed in 66–72 h or later.

### Histopathology of the skin at tick attachment sites on 24 h tick-immune guinea pigs shows increased dermal inflammation

**Figure 5:**
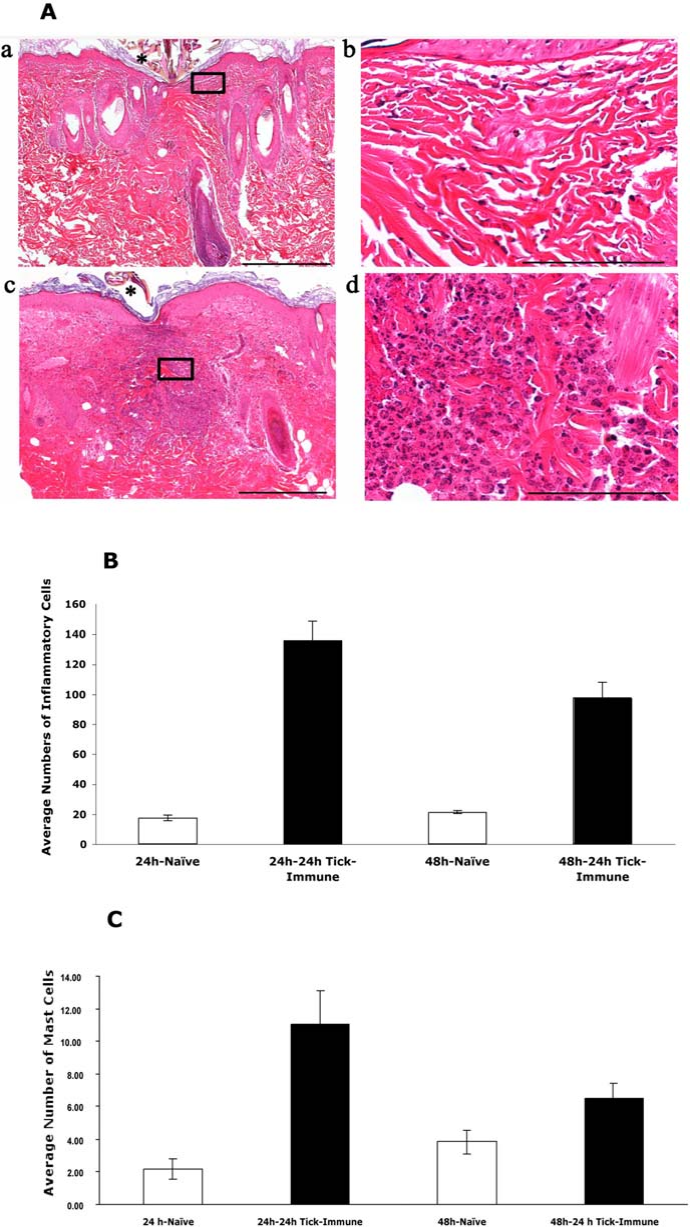
Histopathology of skin punch-biopsies from 24 h tick-immune animals shows increased inflammation. Representative hematoxylin and eosin-stained sections of guinea pig skin punch biopsies obtained at 24 h near tick-attachment sites (*) from naive control (a, b), and 24 hour tick- immune (c, d). Samples from the 24-hour tick-immune animal had the highest number of inflammatory cells (c) which was characterized by a predominance of heterophils (d). In comparison inflammatory infiltrates were markedly reduced and comprised predominantly of mononuclear cells in 24-hour naïve animals (a, b). Scale bar = 500 µm (a, c); Scale bar = 100 µm (b, d). B. Dermal inflammatory cells in skin sections of 24 h tick-immune animals showed a statistically significant (*P<0.001*) increase in the numbers compared to naïve animals. C. Toluidine blue positive cells representing basophils/mast cells also showed a statistically significant increase (*P<0.05*) in skin biopsies of 24 h tick-immune animal compared to naïve animal (Error bars represent±SEM)

Skin samples obtained 24 hours after tick attachment from the 24 h tick-immune animal revealed increased number of dermal inflammatory cells, comprised predominantly of heterophils (Fig 5A, Panels c and d), and inflammation was significantly higher in the 24 h tick-immune samples compared to naïve samples (Fig 5B) (*P<0.001*). Skin biopsies of the 24 h tick-immune animal showed significantly higher number of basophils/mast cells both at 24 and 48 h (Fig 5C) compared to skin samples from a naive animal (*P<0.001*) and was characteristic of cutaneous basophil hypersensitivity [38]. These observations underscore the hypothesis that salivary proteins expressed in the first 24 h of tick feeding play a key role in eliciting tick-immunity in the vertebrate host.

### 24 h tick-immunity impairs Borrelia burgdorferi transmission to the vertebrate host

**Figure 6:**
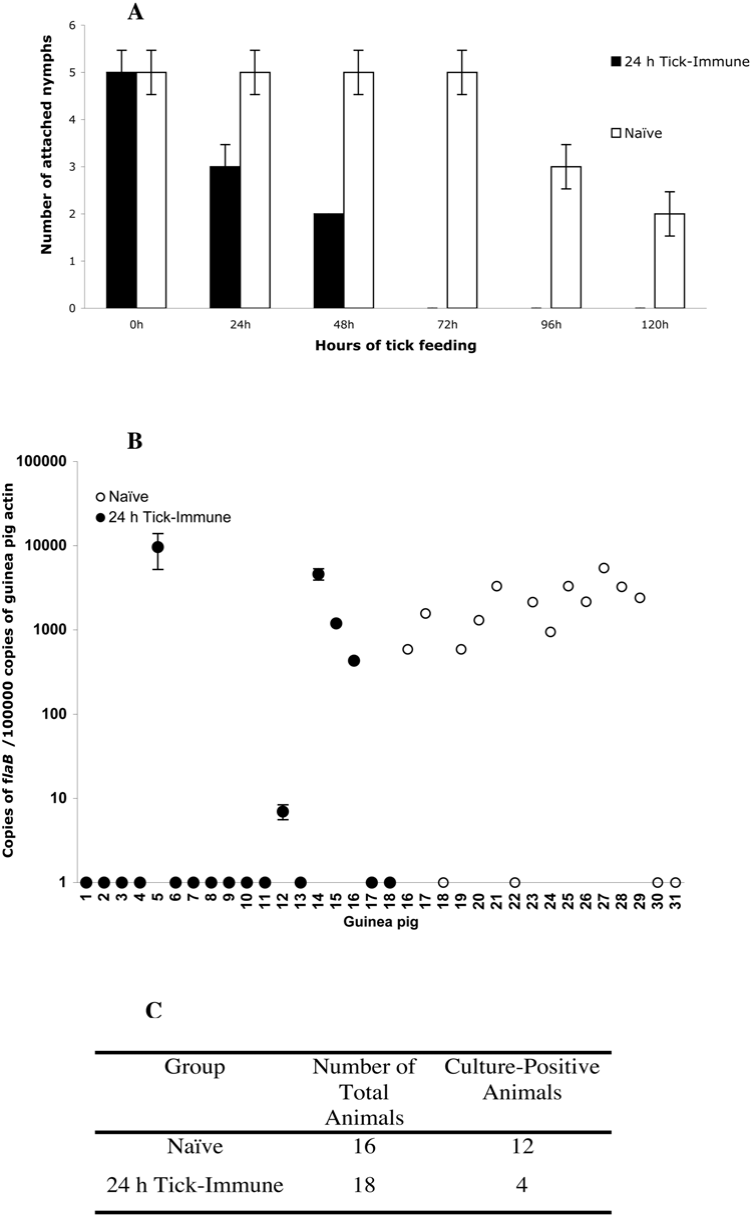
Immunity against 24 h tick salivary proteins impairs *Borrelia burgdorferi* transmission. A. The efficiency of *B. burgdorferi* infected nymphs to feed on 24 h tick-immune animals was significantly decreased (*P<0.02*) compared to that on naïve animals and ticks were rejected within 48 h of attachment. B. Quantitative PCR analysis of guinea pig skin punch-biopsies 4 weeks after tick detachment showed a significant decrease (*P = 0.01*) in spirochete burden in 24 h tick-immune animals compared to naïve animals as evaluated by *B. burgdorferi* *flaB* amplicon levels. C. Culture of skin punch-biopsies in BSK-H medium demonstrated a significant decrease (*P<0.01*) in the number of 24 h tick-immune animals that harbored viable spirochetes when compared to naïve animals. (Error bars represent Average±SD)

Earlier work [37] had shown, using a guinea pig model of tick-immunity, that tick-immunity thwarts *Borrelia* transmission to the vertebrate host. We now examined *Borrelia* transmission in the context of immunity against 24 h tick salivary proteins. 24 h tick-immune guinea pigs were each challenged with 5–6 *B. burgdorferi*-infected *I. scapularis* nymphs. Naïve guinea pigs were similarly challenged and served as controls. At least 20 animals were used in each experimental and control group. Ticks feeding on 24 h tick-immune animals were rejected within 24–48 h and showed decreased engorgement weights (Fig 6A). Four weeks after tick fall-off, RT-PCR analysis of skin punch biopsies obtained from each of the animals also showed that *Borrelia* transmission was significantly decreased (*P = 0.01*) in 24 h tick-immune guinea pigs compared to naïve animals (Fig 6B). Skin punches (obtained from each of the animals two weeks after tick fall-off) when cultured in BSK-H medium for 10 days showed the presence of viable spirochetes in 12 out of 16 animals in the control group while only 4 out of 18 animals (*P<0.01*) in the 24 h tick-immune group showed viable spirochetes (Fig 6C).

### Tick-immunity targets salivary components critical for Borrelia transmission to the vertebrate host

**Figure 7:**
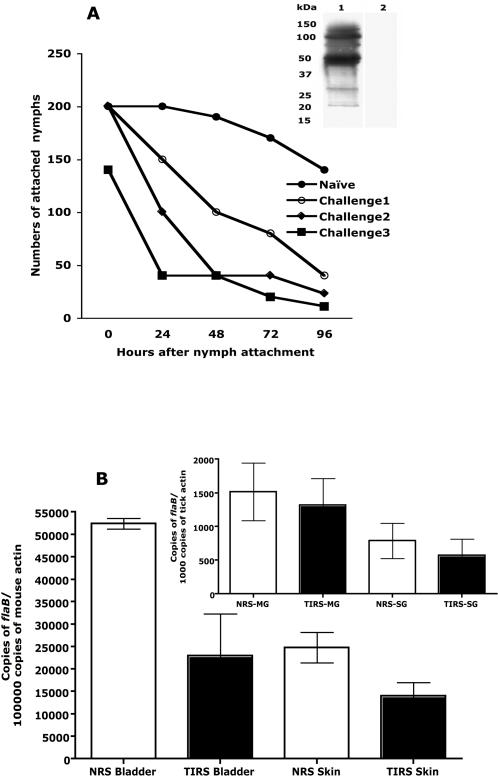
Passive transfer of rabbit tick-immune serum to mice impairs the ability of *B. burgdorferi* infected nymphs to transmit spirochetes to mice. A. The ability of nymphs to feed on rabbits upon repeated infestation was decreased. Inset. Immunoblot of *B. burgdorferi* protein extract probed with anti-*Borrelia* antiserum (lane 1) or nymph-immune rabbit serum (lane 2). B. Inset. Quantitative RT-PCR assessment of *Borrelia* burden as scored by levels of *flaB* amplicons in the midguts (MG) and salivary glands (SG) of nymphs feeding on mice that received rabbit tick-immune serum (RTIS) or naive serum (NRS); and quantitative PCR assessment of *Borrelia* burden in the bladder and skin of mice that received rabbit tick-immune serum (RTIS) or naïve (NRS) serum.

Mice serve as reservoir hosts of *I. scapularis* [1], and do not readily express resistance to tick feeding [39]. Hence a mouse model of *B. burgdorferi* transmission by *I. scapularis* nymphs was utilized to examine the impact of acquired tick-immunity on pathogen transmission without the overlying impact of tick-immunity on tick engorgement. Unlike guinea pigs, rabbits elicited a robust humoral response to tick salivary proteins upon repeated infestations with *I. scapularis* nymphs (Fig 1B) that resulted in rapid rejection of ticks within 24 h of attachment (Fig 7A) and significantly decreased tick engorgement weights on the tick-immune animals (1.0 mg±0.11 SEM) (n = 25) compared to that on control animals (3.79 mg±0.17 SEM) (n = 45) (*P<0.0004*). Nymph-immune rabbit serum was tested on a western blot to confirm that there was no reactivity to *B. burgdorferi* protein extract (Fig 7A, Inset) and then transferred to naïve C3H/HeN mice 24 h prior to placement of *B. burgdorferi*-infected nymphs. Control mice received naïve rabbit serum. The nymphs fed comparably on both groups of mice with no significant differences in engorgement weights on control (1.19 mg±0.19) (n = 25) and on experimental mice (1.09 mg±0.27) (n = 26). The *Borrelia* burden in the midguts and salivary glands of nymphs, as assessed by quantitative RNA-PCR (Fig 7B, Inset), was not significantly different (*P>0.1*) in experimental or control groups. Although all animals in both control and experimental groups got infected, the transmission efficiency of *Borrelia* to mice that received tick-immune sera was decreased when compared to control mice, as evaluated by decreased spirochete burden in the skin (*P = 0.04*) and bladder (*P = 0.05*), 21 days after the ticks had detached (Fig 7B).

## Discussion

*I. scapularis* ticks are obligate hematophagous arthropods that vector several human pathogens. With the goal of developing vaccines to block tick feeding and the ensuing microbial transmission, molecular and immunological approaches [12], [23], [31], [40], [41] have been used to characterize the critical antigens. In 1939 Trager showed that [26] repeated tick infestations provoked guinea pig immunity against *D. variabilis* salivary proteins characterized by cutaneous reactions at tick feeding sites that resulted in tick rejection within 24–48 h of attachment. Studies in numerous vertebrate host species showed that these cutaneous responses, the hallmark of acquired resistance to ticks, were primarily due to a rapid degranulation of mast cells and basophils at the feeding lesion triggered by the engagement of tick-antigen and antigen-specific antibodies to Fc receptors [29], [35], [36]. Upon repeated infestations of vertebrate hosts, antibodies to tick proteins were increasingly elaborated [33], [42], [43] and tick-immune serum passively transferred to naïve animals conferred partial protection from tick challenge, demonstrating a contribution of the humoral response [44], [45]. This phenomenon of acquired tick-immunity has therefore been exploited for several decades to identify proteins critical for feeding in tick saliva and salivary glands, based on their reactivity to tick-immune serum [27], [31], [46], but have provided limited success.

In this study, we demonstrate definitively that the *I. scapularis* transcriptome and proteome is dynamic and present a paradigm shift in the search for salivary proteins critical for establishing tick feeding. While several earlier observations on ixodid ticks suggested that the composition of the tick saliva changes during feeding [47], [48], [49], [50], the paucity of molecular tools to dissect the tick proteome and transcriptome perhaps confounded a detailed analysis and the full importance of these initial observations was not recognized. The *I. scapularis* genome is being sequenced [51], and the transcriptome of fed nymphs and adults partially cataloged [10], [11]. We utilized this partial database [10] and generated an oligonucleotide array representing a subset of genes that encode for secreted tick salivary proteins. Since most of the genes represented in this array correspond to genes identified from fed nymphs and adults, it was not surprising that most of the genes were not expressed or expressed at lower levels in 24 h fed salivary glands compared to that in 66 h fed salivary glands. At this juncture, in the absence of whole genome information, it is not possible to infer the physiological significance of the changing transcriptome. The observation that the transcriptome of *I. scapularis* salivary glands contained structural paralogs of several genes suggested that paralogy might offer a potential strategy to evade host immunity. It was reasoned that different paralogs would be expressed at different stages of feeding. However, the expression levels of most of the *salp14* paralogs were not dramatically altered during feeding and, the expressions of AF483703, AF483681 that are paralogs of AF483786 appeared to be increased at 66 h of feeding (Table 1). Cross-hybridization of transcripts corresponding to different structural paralogs on arrays cannot be ruled out and careful design of PCR primers will be required to assess the expression profiles of individual paralogs. Importantly, this sub-set mini-array analysis has demonstrated that the tick transcriptome at 24 (early phase) and 66–72 h (late phase) of feeding is different (Table 1, Fig 3). Consistent with the observations made using the mini-array, the proteome analysis by DIGE and HPLC analysis iterated that the salivary gland proteome changes during feeding (Fig 1 and 2). It is well established that tick feeding proceeds in different phases [52], hence, it is logical to expect that the different phases of feeding may be driven by concomitant changes in the expression profile of tick salivary genes and therein perhaps, lies the essence of successful tick feeding.

Since acquired tick-immunity resulted in rejection of ticks within the first 24–48 h of feeding, we hypothesized that tick-immunity must be directed against proteins expressed early (in the first 24 h) during engorgement. Underpinning this assumption was the observation that immunization with salivary gland extracts from 66 h fed nymphs did not provoke immunity to ticks (Fig 4A). Further, protein fractions from the 24 and 66 h salivary glands showed differential reactivity with rabbit tick-immune serum (Fig 1B and 2B) and 24 h salivary fractions reacted more avidly than the 66 h fractions. Interestingly, Dharampaul et al [47] had observed that the antigenic composition of *A. hebraeum* ticks changed with engorgement size and tick-immune serum against *A. hebraeum* readily reacted with salivary gland extracts from ticks with smaller engorgment size.

We recognize that immunization of guinea pigs with 24 h salivary gland extracts would require a vast number of ticks to generate sufficient protein, standard immunization regimens requiring up to 100 µg of protein/animal. We therefore tested our hypothesis using an alternative strategy wherein ticks were allowed to repeatedly feed for 24 h to ensure that the animal was exposed only to antigens expressed in the first 24 h of feeding and demonstrate for the first time that immunity directed against proteins expressed in the first 24 h of feeding elicits all the hallmarks of acquired tick-immunity including redness, early tick rejection and decreased engorgement weights (Fig 4). Histopathology of skin biopsies from 24 h tick-immune animals demonstrated cellular infiltrates composed predominantly of mast cells and basophils (Fig 5). This was consistent with earlier observations made on tick-immune animals that showed the sequence of increasing cellular infiltrates and invoked the role of degranulated mast cells, eosinophils and basophils in mediating the immediate type hypersensitivity associated with rapid tick-rejection within the first 24 h of attachment on tick-immune animals [29]. Taken together, these results obtained using a guinea pig model of tick-immunity against *I. scapularis* ticks suggest that antigens expressed in the first 24 h of tick feeding are key players in expressing tick-immunity in the vertebrate host and represent therefore proteins critical to establish the onset of feeding. We anticipate that this finding when extended in future studies to other tick species and to other animal-models of tick-immunity will fuel the discovery and design of novel anti-tick vaccines.

The major enthusiasm with acquired tick-immunity stems from the earlier observations that ticks feeding on tick-immune animals are not able to transmit pathogens efficiently [46], [53], [54] and is underscored by a recent study that residents of Lyme disease-endemic areas who have had multiple exposures to uninfected ticks may also be protected from Lyme disease [55]. Nazario et al [37] showed that ticks remained attached to tick-immune guinea pigs for longer than 24 h but were unable to efficiently transmit *Borrelia*. Earlier, Piesman's work [56] had shown that *B. burgdorferi*-infected *I. scapularis* ticks can transmit spirochetes to a naïve vertebrate host if they remain attached for longer than 24–36 h on the host; this time essential for the replication and migration of spirochetes from the midguts to the salivary glands from where they exit the tick vector [6]. Nazario et al's [37] observation suggested that tick-immunity may impair pathogen transmission by additionally targeting tick proteins critical perhaps for *Borrelia* growth in the ticks and its migration to the salivary glands for transmission. It was therefore important to examine if immunity directed against proteins expressed in 24 h would be sufficient to also impair *Borrelia* transmission.

Using a guinea pig model of *B. burgdorferi* transmission, we evaluated whether immunity directed against the 24 h salivary gland proteins impaired *Borrelia* transmission. Similar to Nazario's observations, although *Borrelia*-infected ticks fed poorly on 24 h tick-immune guinea pigs, but at least some remained attached up to 48 h (Fig 6A), providing sufficient time for spirochete transmission to the vertebrate host. However, the ability of *Borrelia*-infected nymphs to transmit spirochetes to 24 h tick-immune guinea pigs was drastically impaired (Fig 6B and 6C).

Unlike guinea pigs, rabbits provided a robust humoral response to tick salivary proteins upon repeated infestations with *I. scapularis* nymphs (Fig 1B) that resulted in rapid tick rejection within 24–48 h of attachment, as shown in a representative animal (Fig 7A) and impaired tick engorgement as observed in guinea pigs (Fig 4D). Also, consistent with the hypothesis that tick-immunity is directed against salivary proteins expressed in the first 24 h of tick attachment, tick-immune rabbit sera reacted readily with 24 h tick salivary proteins (Fig 1B and 2B). Thus, although, nymphs were allowed to feed to repletion on the rabbits, rabbit tick-immunity may also be predominantly directed against 24 h salivary antigens. We therefore transferred *I. scapularis* nymph-immune rabbit serum to experimental C3H mice and naïve rabbit serum to control C3H mice and then challenged with *Borrelia*-infected *I. scapularis* nymphs. The engorgement weights of ticks and *Borrelia* burdens in the midguts and salivary glands were comparable in both control and experimental groups. Interestingly, *Borrelia* transmission efficiency of ticks feeding on mice that received tick-immune serum was significantly decreased when compared to ticks feeding on control mice (Fig 7B). An earlier study by Wikel et al [39] showed that ticks feeding on mice repeatedly infested by *I. scapularis* nymphs were able to feed successfully, but were not able to transmit *Borrelia* efficiently. It is also interesting to note that in our recent study, Salp15, a tick salivary protein, facilitated the transmission of *B. burgdorferi* to mice [9] and reacted readily with rabbit tick-immune serum [12]. Ablation of *salp15* expression impaired *Borrelia* transmission to mice but did not alter the ability of ticks to engorge on mice and did not impair spirochete growth and migration within ticks [9]. These observations gather evidence in favor of the premise that acquired tick-immunity may target additional events redundant for feeding but critical for pathogen transmission and provokes the possibility that immunity against tick proteins critical for transmission can serve as a novel approach to block microbial transmission.

The passive transfer experiment suggested that tick feeding and pathogen transmission might require different salivary proteins. It is also likely that salivary proteins critical for enabling tick feeding on rabbits/larger mammals may indeed be different from proteins critical for feeding on mice. That the tick transcriptome may change not only during feeding, but also on different hosts has been suggested by Nuttall's earlier work on *Rhipicephalus appendiculatus* ticks [50] and is underscored by our observations.

These results provide biological evidence that immunity against 24 h salivary proteins expresses all the characteristics of and is indistinguishable from tick-immunity expressed by natural repeated tick-infestations. The current study shifts the focus from late phase proteins and provides evidence that proteins expressed within 24 h of feeding play a critical role in establishing the early phase of tick-host interaction and enabling pathogen transmission. Defining these proteins will be the next step that will reveal how these proteins may function in the initial events that allow the vector to engage with the host and why these events also determine the success of *B. burgdorferi* transmission. The last decade has been a turning point for tick genomics with concerted efforts from various research groups promoting not only an increasing knowledge of tick genes and proteins but also providing novel molecular techniques to examine their functions [51], [57]. The field now stands poised, to better identify these 24 h tick salivary proteins and determine their role in establishing feeding and in pathogen transmission.

## Materials and Methods

### *I. scapularis* ticks

*I. scapularis* nymphs and larvae were obtained from a tick colony at the Connecticut Agricultural Experiment Station (New Haven, CT).

### B. burgdorferi-infected mice and nymphs

A low-passage-number clonal isolate of *B. burgdorferi* N40 that is infectious to mice [58] was used to inoculate C3H mice. Roughly, 100 µl of 1×10^5^ N40 spirochetes/ml was injected subcutaneously. Skin punch biopsies were collected from each mouse 2 weeks after inoculation and DNA isolated using the DNeasy kit (QIAGEN, Valencia, CA) and tested by PCR for the presence of spirochetes as described below. *I. scapularis* larvae were placed on *B. burgdorferi*-infected C3H mice and fed larvae molted to generate *B. burgdorferi*-infected nymphs.

### 2D protein analysis

A qualitative analysis of the *I. scapularis* salivary gland proteome was carried out by Differential 2D Fluorescence Gel Electrophoresis (DIGE) at the W.M Keck Facility at Yale University. Salivary gland extracts from 200 *I. scapularis* nymphs fed for 24 and 66 h were suspended in a cell lysis buffer (7M urea, 2M thiourea, 4% CHAPS, 25 mM Tris, pH 8.6 at 4°C) and protein concentration estimated by amino acid analysis at the W.M Keck Facility at Yale University. Equal amounts of protein (50 µg) from 24 and 66 h salivary gland extracts were then differentially labeled *in vitro* with Cy3 and Cy5 N-hydroxysuccinimidyl ester dyes as described in the Ettan DIGE manual (GE Healthcare, NJ). A third dye (Cy-2) as an internal (pooled 25 µg of 24 h+25 µg 66 h salivary gland extracts) standard to permit normalization of multiple gels and for internal normalization was also included. Rehydration Buffer was added to a total volume of 400 microliters, and isoelectric focusing was carried out in the first dimension on 24 cm Immobiline (IPG) Drystrips (GE Healthcare, NJ) using a pH 3–10 range, and a 12.5% polyacrylamide gel in the second dimension. The gel was then sequentially scanned at all three wavelengths using the Typhoon 9410 Imager (GE Healthcare, NJ) and images exported into the DeCyder (GE Healthcare, NJ) software package to assess differentially expressed protein spots. The DeCyder software automatically outputs a listing of significant differences in protein expression including t-test values, using the Cy-2 internal standard. The protein spots were not excised for identification.

### Fractionation of salivary gland extracts by High performance Liquid Chromatography (HPLC)

Salivary gland protein extracts from nymphs fed for 66 and 24 h were suspended in water and 50 µg of each protein extract in a volume of 50 µl was loaded on a 1 mm×25 cm Vydac C-4 (5 micron particle size, 300 pore size) reverse-phase column and fractionated on a Hewlett Packard 1090 HPLC system equipped with an Isco Model 2150 Peak Separator. The column was equiliberated with 98% buffer A (0.06%TFA) and 2% Buffer B (0.052% TFA, 80% acetonitrile). The protein was eluted at 5 µl/min with the following gradient program: 0–60 min (2–37% B), 60–90 min (37–75% B) and 90–105 min (75–98% B) and fractions detected by their absorbance at 210 nm and collected in Eppendorf tubes.

### Array construction

All *I. scapularis* protein sequences in the NCBI database (www.ncbi.nlm.nih.gov) (as of the start of the experiment) were downloaded into a zipped file and inserted into a Microsoft Access database. Irrelevant sequences were parsed out to retain only *I. scapularis* sequences. A Visual Basic macros embedded in the Access database was then used to pass each sequence through WoLF PSORT (located at http://wolfpsort.seq.cbrc.jp) to identify cellular localization of the protein; and the Signal P 3.0 server (www.cbs.dtu.dk/services/signalP/) to identify if the proteins are secreted. Output from these programs was recorded and the data exported to Microsoft Excel. The FASTA files of the nucleotide sequences for membrane and secreted proteins were processed twice using the program ArrayOligoSelector 3.8.1(http://.sourceforge.net/projects/arrayoligosel) to generate unique 50 mers corresponding to the 3′ and 5′ ends of the gene. Since the genome sequence of *I. scapularis* is not available, we bear in mind that the oligo's selected by the ArrayOligoSelector, may have similarities to sequences in the genome that are not yet identified. Several paralogous gene families have been identified in the *I. scapularis* salivary gland transcriptome with very high levels of polymorphisms in certain groups [11]. We did not include all members of a paralogous family, especially if they differ by very few nucleotides among themselves. An arbitrary cut off of 80% identity was set to exclude representation of members of a family that are 80% or more identical to each other. The output from the ArrayOligoSelector was also analyzed using BLAST 2.2.9 to ensure specificity. The oligonucleotides synthesized at 100 µM concentration (Sigma Genosys, MO) were diluted to a concentration of 5 µM in 50% DMSO and maintained in 96 well plates. Using a Beckman Biomek FX robotic liquid handler the 96-well plates were condensed to 384–well plates containing the diluted oligonucleotides. The DNA from these plates was printed onto 25 mm×75 mm UltraGAPS Coated slides (Corning, NY) using a BioRad VersArray Pro Micoarrayer (BIO-RAD Laboratories, CA). The oligonucleotides were immobilized, by UV cross-linking at 2000 mJoules using a Stratalinker (Stratagene, CA). The oligonucleotides corresponding to each gene was spotted in triplicate, to increase precision of spot intensity measurements [59].

### Array hybridization

Approximately 20–25 nymphs were placed on each naïve mouse and allowed to feed for 24 or 66 hours. Fed ticks were removed, dissected to remove salivary glands and total RNA isolated as described [60] and pooled in groups of 20 ticks separately. At least 4 separate pools of biological triplicates for each 24 h and 66 h samples were generated and RNA quantity assessed by spectrophotometry. Equal amounts of RNA from 24 and 66 h fed salivary gland samples were amplified using the Amino Allyl MessageAmp aRNA Amplification and Labeling kit (Ambion Inc, Austin TX). This procedure utilizes an aRNA amplification procedure developed by Van Gelder [61] and does not significantly skew the representation of individual mRNA species in the RNA population[62], [63]. The amplification was conducted in the presence of amino allyl UTP so as to incorporate the aaUTP into the aRNA. The aRNA was purified and quantified by spectrophotometry. About 5 µg of aRNA prepared from 24 h and 66 h fed salivary gland RNA was used for labeling with amine reactive dyes Cy3 and Cy5 (CyDye Post-Labeling Reactive Dyes, Amersham Biosciences, NJ) respectively using the protocol described in the aRNA Amplification and Labeling kit (Ambion Inc, TX). In parallel we also set up a technical replicate in which the dyes were switched/swapped between each set of 24 and 66 h samples to overcome bias due to the dye itself [64]. This experimental set-up included 4 biological replicates with at least 2 dye-swap technical replicates. The array slides were pre-hybridized (5xSSC, 5XDenhardts, 1%SDS and 0.1 µg/ml salmon sperm DNA) for 1h at 42°C and hybridized to the aRNA probe in fresh pre-hybridization solution containing 50% formamide overnight at 42°C. The slides were then washed as described in the UltraGAPS Coated Slides instruction manual (Corning, NY). The washed slides were scanned on a GenePix 4000A scanner and data manipulated with GenePix software (Axon Instruments, CA). The data were then first quantile normalized [65] and significance of differential expression was then assessed with the Wilcoxon rank sum test [66]

### Array validation by quantitative RT-PCR

Ten genes that were differentially expressed at 24 and 66 h as observed by the array analysis were selected for validation by quantitative RT-PCR. RNA was prepared from pools of 5–6 nymphal salivary glands isolated from nymphs fed for 24 and 66 h on C3H/HeN mice as described above and cDNA was synthesized using the iScript RT-PCR kit (BIORAD, CA). At least 4 pools were examined. cDNA was analyzed by PCR for the expression of selected genes (indicated by their GenBank Accession numbers) using the following primer pairs: AF483728: 5′gatgcctccaataaaccact3′; 5′ccgacggagaagaggaat3′; AF483712: 5′aacggcgccctcaatcca3′: 5′gcagccgaaaaagcagacacc3′; AF483686: 5′gacaaccttgcaatcccctaca3′: 5′gagcgcgtcggcaataatct3′; AF483737: 5′attgggggcttcattcttg3′: 5′agtcacttgggccgcttctc3′; AF209921: 5′agaccaaatcatgggacc3′: 5′cagtgggcgcaggagtatag3′; AF209918: 5′gctccccctgaagaagaccc3′:5′gcgtcgtgttgcaatattc3′; AF278575: 5′gagctcggatggcgaaccaac3′:5′ggtaccgcaatcctcaagtgtg3′; AF209912: 5′attccaacccgcttatctc3′:5′aggcattgctggacaca3′; AF483729: 5′acggcgagctttgcggtcttt3′:5′gttggattcagcggcgttttcttc3′; AAY333959: 5′gctctcggacttcaacaagc3′: 5′cgtgcttctccacaaactg3′; AAY66642: 5′tcgtctcatttgcgctcttctg3′: 5′tttcccgatgctaccgtcaca3′. Quantitative PCR was performed using the iQ Syber Green Supermix (Biorad, CA) on a MJ cycler (MJ Research, CA). Data was normalized to tick actin and fold change in gene expression in 66 h salivary glands estimated relative to the levels in 24 h salivary glands or *vice versa* using the equation 2^−ΔΔCt^ [67].

### Tick-immune guinea pigs

To generate guinea pigs immune to tick salivary antigens expressed specifically in 24 h fed nymphs, 30 pathogen-free nymphs were placed on 6–8 week old female guinea pigs and ticks allowed to attach and feed for 24 h and deliberately removed. The animals were allowed to rest for 2 weeks and the process of 24 h infestation repeated 2 more times with 30 pathogen free nymphs on each animal. Age-matched female guinea pigs maintained without tick infestations served as the naïve control group. At least 4 animals were used in each group in individual experiments and the experiment was repeated at least 4 times. Tick-immune guinea pig serum was collected after the 4^th^ challenge by retro-orbital bleed or by cardiac puncture using protocols approved by the Yale Animal Care and Use Committee. To immunize guinea pigs with salivary gland extracts (SGE) from engorged nymphs, at least 3 age-matched female guinea pigs (6–8 weeks old) were immunized subcutaneously with 30–50 µg of SGE (isolated from nymphs fed to repletion on guinea pigs) in incomplete Freunds Adjuvant (IFA) in 4 to 5 sites along the flank and boosted at 14 and 28 days with same amounts of SGE in IFA. Complete Freunds Adjuvant (CFA) was avoided as it resulted in immunization sores. Immune sera was collected and analyzed for reactivity to engorged nymph salivary gland extracts by routine western blot analysis as described below. The experiment was conducted a least three times.

### Tick feeding

In experiments to assess the impact of 24 h tick-immunity on tick feeding, 30 pathogen-free nymphs were placed on each naïve and 24 h tick-immune guinea pigs and allowed to feed to repletion. The animals were monitored everyday for redness and erythema at tick feeding sites, tick numbers and tick engorgement weights recorded as and when the ticks detached. The impact of immunization with 66 h SGE on tick feeding was also performed as above.

### Tick-immune rabbit

To generate tick-immune rabbits at least 100 clean I. scapularis nymphs were placed on each ear of 4–5 week old female New Zealand white rabbits and ticks allowed to attach and feed to repletion. The animals were allowed to rest for 2 weeks and the process of infestation repeated 3 more times. Age-matched rabbits maintained without tick infestations served as the naïve control group. At least 4 animals were used in each group. Tick-immune rabbit serum was collected after the 4^th^ challenge by ear-bleed or by cardiac puncture using protocols approved by the Yale Animal Care and Use Committee. This serum was tested for reactivity to tick salivary gland extracts by western blot analysis as described below. An immunoblot of cultured Borrelia (N40) total protein extracts (5 µg) was also probed with the rabbit nymph-immune sera by routine western blot analysis to test for reactivity to B. burgdorferi. Rabbit anti-Borrelia serum generated earlier in the lab [9] served as a positive control.

### Histology of skin punch-biopsies

Three, 3 mm punch tissue biopsies per animal were fixed in Zenkers solution (Fisher Scientific, MA) and transferred to 70% ethanol, processed (Excelsior Processor; Thermo Electron Corporation, Pittsburgh, USA), embedded in paraffin (Blue Ribbon, Surgipath Medical Industries, Inc., Richmond, USA), and serially sectioned (20 slide each) at 5 microns. The 1st, 5th, 10th, 15th, 20th slides were stained with hematoxylin and eosin (HE) and the 9th and 14th stained with toluidine blue (T-Blue) by routine methods to evaluate the severity of inflammation (HE) and mast cell/basophil (T-blue) infiltrates by microscopic examination. Observers were blinded to the study conditions until after the histopathologic features were assessed. To semiquantitatively assess the number of mast cells/basophils within the dermis, the entire tissue field was examined at 40× magnification and all cells with purple (basophilic) cytoplasmic granules recorded for each tissue section on the T-Blue- stained slides for a total of 12 sections of tissue examined per animal. To assess the severity of inflammation (macrophage, lymphocyte, heterophil) within the dermis, a semiquantitative method [68] was used where inflammatory cells were counted manually on a Denominator laboratory counter (The Denominator Co, Inc., Woodbury, CT) for a 100 square (1 mm×1 mm) grid (KR-406B, Klarmann Rulings Inc. Litchfield, NH) for each of three fields at 40× for one each of the three tissue sections per slide. Thus, a total of 5 sections per punch biopsy, 15 sections per animal and 45, 100 square grid areas were evaluated per animal. The nature of the inflammatory infiltrate was classified as predominantly mononuclear, predominantly heterophilic or mixed. Digital light microscopic images were recorded using a Zeiss AxioScope microscope, AxioCam MRC Camera and AxioVision 4.5 imaging software (Carl Zeiss Microimaging, Inc., Thornwood, NY, USA), and optimized in Adobe Photoshop 8.0 (San Jose, CA).

### B. burgdorferi transmission

In experiments to address *Borrelia* transmission to guinea pigs, 5–6 *B. burgdorferi* (N40) infected nymphs were placed on each 24 h tick-immune and experimental guinea pig (at least 4 animals in each group) and allowed to feed to repletion. The experiment was performed at least 4 times. After tick detachment, transmission was assessed by culture and by quantitative PCR of skin punches at 2 and 4 weeks respectively.

### Quantitative PCR to estimate spirochete burden

Guinea pig skin punch biopsies were obtained from sites near and distal to tick attachment sites at 2 and 4 weeks respectively, after tick engorgement and suspended in DNAeasy suspension buffer (QIAGEN, CA) and processed for DNA isolation according to the manufacturer's protocol. The resultant DNA was analyzed by quantitative PCR using the iQ Syber Green Supermix (Biorad, CA) on a MJ cycler (MJ Research, CA) for the presence of *Borrelia* using *flaB* primers (provided below) and results normalized using *actin* primers as shown. Data were analyzed using Microsoft Excel software. A two-tailed Student's *t*-test was used to analyze qPCR data. A *P* value <0.05 was considered to be a significant difference.

### Culture

Guinea pig skin punch biopsies were cleaned with Betadine, suspended in 10 ml of complete BSK-H medium (Sigma-Aldrich, St Louis, Mo) and spirochetes allowed to grow for 12–14 days at 30°C. The spirochetes were then visualized under a dark-field microscope (Axiostar, Zeiss) and scored for presence or absence of spirochetes.

### Passive transfer of tick-immune serum to mice

Serum obtained from tick-immune rabbits were passively transferred by intraperitoneal inoculation into C3H/HeN mice 24 h prior to placement of *B. burgdorferi*-infected nymphs. Control mice were inoculated with 500 µl of naïve rabbit serum. At least three mice were used in each group. At least 5–6 *B. burgdorferi*-infected nymphs were placed on each mouse and ticks allowed to feed to repletion. Tick engorgement weights were recorded as before and tick salivary glands and midguts dissected for RNA isolation as described below

### Tick RNA isolation and RT-PCR

The nymphs fed on control and experimental animals were dissected and salivary glands (in pools of three pairs) and midguts (in pools of 2) suspended in Trizol and RNA isolated according to the manufacturer's protocol (Invitrogen, CA). cDNA was synthesized using the iScript RT-PCR kit (BIORAD, CA) and analyzed by PCR for the expression of tick *actin* and *flaB* genes using the primers listed: tick actin F-5′ggcgacgtagcag 3′ and tick actin R-5′ggtatcgtgctcgactc 3′; flaBF-5′ttcaatcaggtaacggcaca 3′ and flaBR-5′gacgcttgagaccctgaaag 3′. Quantitative PCR was performed using the iQ Syber Green Supermix (Biorad, CA) on a MJ cycler (MJ Research, CA). Data were analyzed using Microsoft Excel software. A two-tailed Student's *t*-test was used to analyze the data and a *P* value <0.05 was considered to be a significant difference.

### Western Blot and Dot-Blot analysis to assess differential reactivity to salivary gland extracts

Salivary glands were isolated from 24 and 66 h fed nymphs. The tissues were suspended in sterile PBS (100 µl of PBS per 50 salivary gland pairs) and homogenized. Total protein was quantified by the Bradford method. Equal amounts of salivary gland protein (2 µg) from 24 and 66 h fed ticks were electrophoresed on an SDS 4–20% gradient polyacrylamide gel, transferred to nitrocellulose membranes and processed for immunoblotting. The immunoblots were incubated separately with nymph-immune rabbit sera or with naïve rabbit sera. Bound antibodies were detected by using horseradish peroxidase-conjugated goat anti-rabbit secondary antibodies (Sigma–Aldrich, St. Louis, MO). The immunoblots were developed using a Western Lightening chemiluminescence kit (Perkin Elmer Life and Analytical Sciences Inc, Wellesley, MA). For analysis of guinea pig humoral response to immunization with 66 h SGE, fed tick extracts were electrophoresed as described above and incubated with guinea pig anti-66 h SGE sera. Bound antibodies were detected by using alkaline-phosphatase-conjugated goat anti-guinea pig antibody (Sigma-Aldrich, MO) and the phosphatase substrate, NBT-BCIP (Kirkegard and Perry, MD). For analysis of the HPLC fractions of 24 and 66 h salivary gland extracts, equal percentages of each of the fractions were lyophilized to dryness on a DNA110 Speedvac (Savant Instruments, NY), resuspended in 10 µl of water and spotted on to 0.2 µm nitrocellulose membrane (Bio-Rad, CA). The membranes were air-dried and processed as described above for the western blots using nymph-immune rabbit sera.
